# Peripheral Inflammatory Superior Mesenteric Artery Aneurysm Diagnosed by Intraoperative and Histological Findings: A Case Report

**DOI:** 10.3400/avd.cr.20-00078

**Published:** 2020-12-25

**Authors:** Yasuo Suehiro, Hiroyuki Seo, Yuko Kubota, Shigefumi Suehiro, Hidekazu Hirai

**Affiliations:** 1 Department of Cardiovascular Surgery, Osaka Saiseikai Noe Hospital, Osaka, Osaka, Japan

**Keywords:** inflammatory aneurysm, peripheral, superior mesenteric artery

## Abstract

Although rare, superior mesenteric artery aneurysms (SMAAs) are life-threatening due to their high rupture rate. We herein report a case involving an 80-year-old man who presented with acute cholecystitis and who was incidentally found to have a 36-mm peripheral SMAA. A surgical intervention was performed, involving resection of the SMAA and reconstruction of the superior mesenteric artery (SMA) using an autologous vein graft. Intraoperative and histological findings indicated an inflammatory aneurysm, and the postoperative course was uneventful. We believe that resection of the aneurysm and reconstruction of the SMA is the preferred procedure for SMAAs to maintain adequate mesenteric circulations.

## Introduction

Superior mesenteric artery aneurysms (SMAAs) are rare and account for only 6.9% of all visceral arterial aneurysms,^1)^ usually affecting the proximal ≤5 cm of the artery.^2)^ They are often asymptomatic and diagnosed incidentally but are life-threatening due to their high rupture rate. Although several etiologies of SMAAs exist, including infection, atherosclerosis, and arterial dissection, the so-called inflammatory aneurysms are extremely uncommon. Moreover, a standard of care for surgical intervention for SMAAs, including simple ligation, aneurysmectomy and reconstruction, and endovascular repair, has not been established. We herein describe a case of an isolated peripheral inflammatory SMAA treated with aneurysmectomy and reconstruction of the superior mesenteric artery (SMA) using an autologous vein graft.

## Case Report

**Figure 1:**
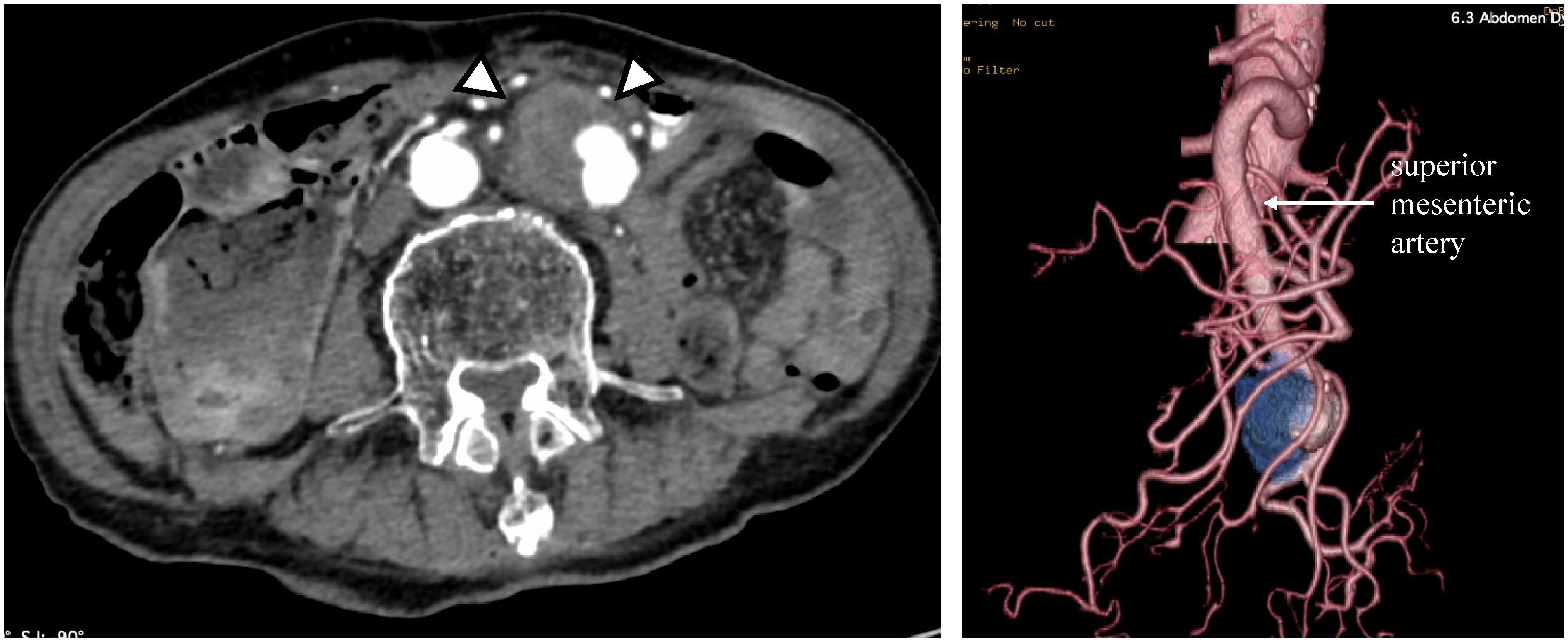
Fig. 1 Contrast-enhanced computed tomography findings. A 36-mm superior mesenteric artery aneurysm (arrow head) was located 11 cm distal to the origin of superior mesenteric artery and contained an intramural thrombus.

An 80-year-old man was admitted to our hospital after presenting with high fever and right subcostal pain. He had no history of trauma, bacteremia, or risk factors for cardiovascular disease such as hypertension, diabetes mellitus, or hyperlipidemia. Physical examination showed that tenderness was present in the right hypochondriac region and a pulsatile mass was palpable in the periumbilical region. Laboratory tests revealed elevated hepatobiliary enzymes, a high serum C-reactive protein (CRP) concentration of 22.1 mg/L, a high white blood cell (WBC) count of 10,100/µL, and a high erythrocyte sedimentation rate of 29 mm/h; however, blood cultures were negative. Additionally, the patient had a normal serum immunoglobulin G4 concentration and negative autoantibodies, which include antinuclear antibody and antineutrophil cytoplasmic antibody (ANCA) serology. Systemic contrast-enhanced computed tomography (CT) revealed enlargement of the gallbladder with wall thickening without stones and a 36-mm dilatation of the SMA. No other vascular entities were detected. Based on these clinical and imaging findings, the patient was diagnosed with acute cholecystitis and SMAA. The SMAA was located 11 cm distal to the origin of the SMA and ended at the origin of the ileocolic artery. No signs of irregular or lobulated arterial walls, soft tissue inflammation or masses around vessels, perivascular fluid collections, or aneurysms with intramural air were noted. The SMAA contained a large intramural thrombus with no findings of occlusion throughout the SMA (**Fig. 1**). Although we considered that infection might have caused the SMAA, CT images showed no typical findings of a mycotic aneurysm. Therefore, we scheduled a surgical intervention for the SMAA after improvement of the cholecystitis, and the patient immediately underwent percutaneous transhepatic gallbladder drainage and intravenous antibiotic therapy. Additionally, since these treatments started, we had performed abdominal CT every 2 or 3 days for following up of cholecystitis and SMAA while strictly monitoring the laboratory data, including WBC count, CRP levels, and fever. After 2 weeks, his condition and clinical findings had remarkably improved. Laboratory tests showed a normal WBC count and serum CRP concentration of 2.68 mg/L. His symptoms, such as high fever and subcostal pain, were diminished. During this period, no growth of the SMAA was found on CT images. Thus, we performed an elective surgical intervention for the SMAA.

**Figure 2:**
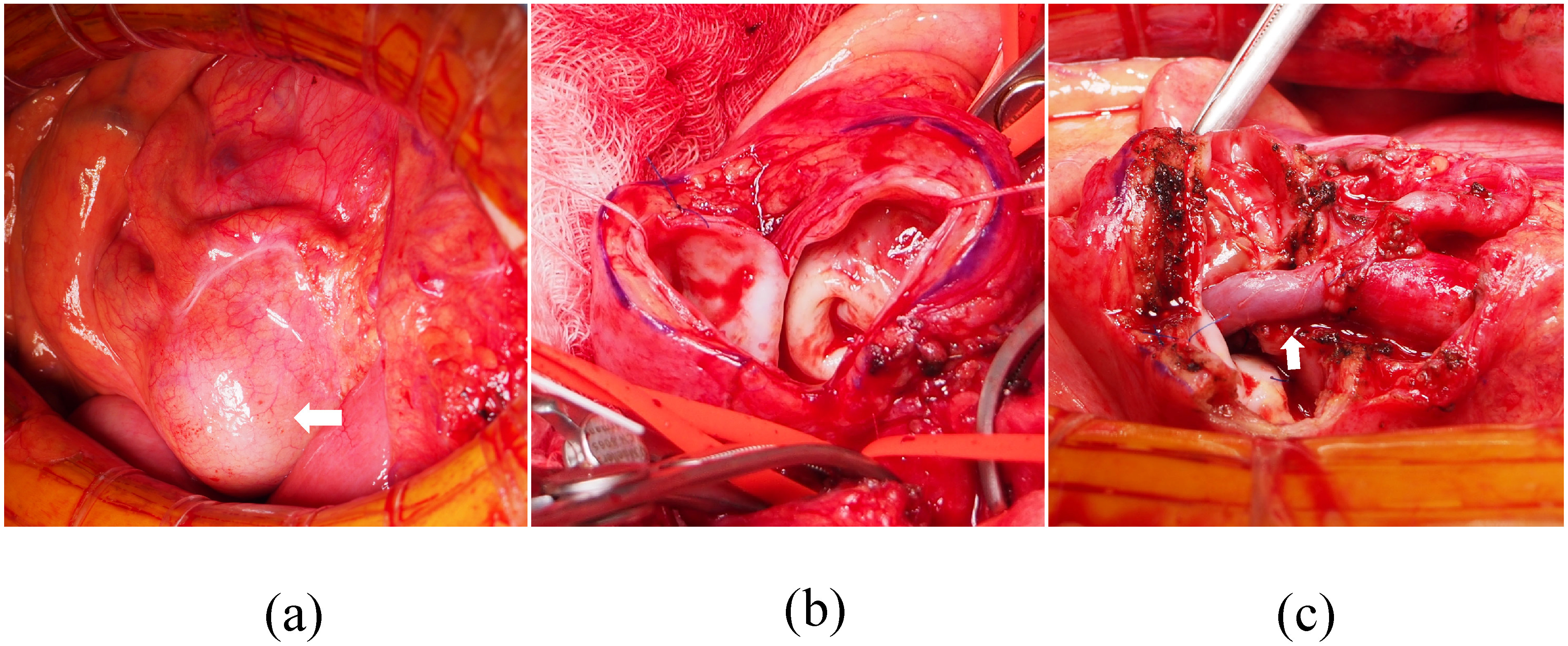
Fig. 2 Intraoperative findings. (**a**) The aneurysm surface was white and glistening (white arrow), and severe adhesion was present between the aneurysm and surrounding tissues. (**b**) The aneurysm wall was very thick. (**c**) We resected the aneurysm and reconstructed the superior mesenteric artery with a saphenous vein graft (white arrow).

The patient was in supine position during surgery, and the SMAA was exposed through an upper median abdominal incision. The surface of the aneurysm was white and glistening, and severe adhesion between the aneurysm and surrounding tissues was present. Although unswollen, the gallbladder had a thickened wall, and there were slight adhesions around it. Tissues between the gallbladder and SMAA were intact. After clamping the proximal and distal sides of the aneurysm, we assessed the viability of the intestinal tract based on intestinal color changes. The aneurysm was subsequently opened. The aneurysm wall was very thick, and a red-brown thrombus was found. A small amount of backflow from the peripheral SMA was present. We resected the aneurysm and interposed a great saphenous vein graft taken from the left groin within the SMA (**Fig. 2**). There were no signs of intestinal tract ischemia before or after reconstruction.

**Figure 3:**
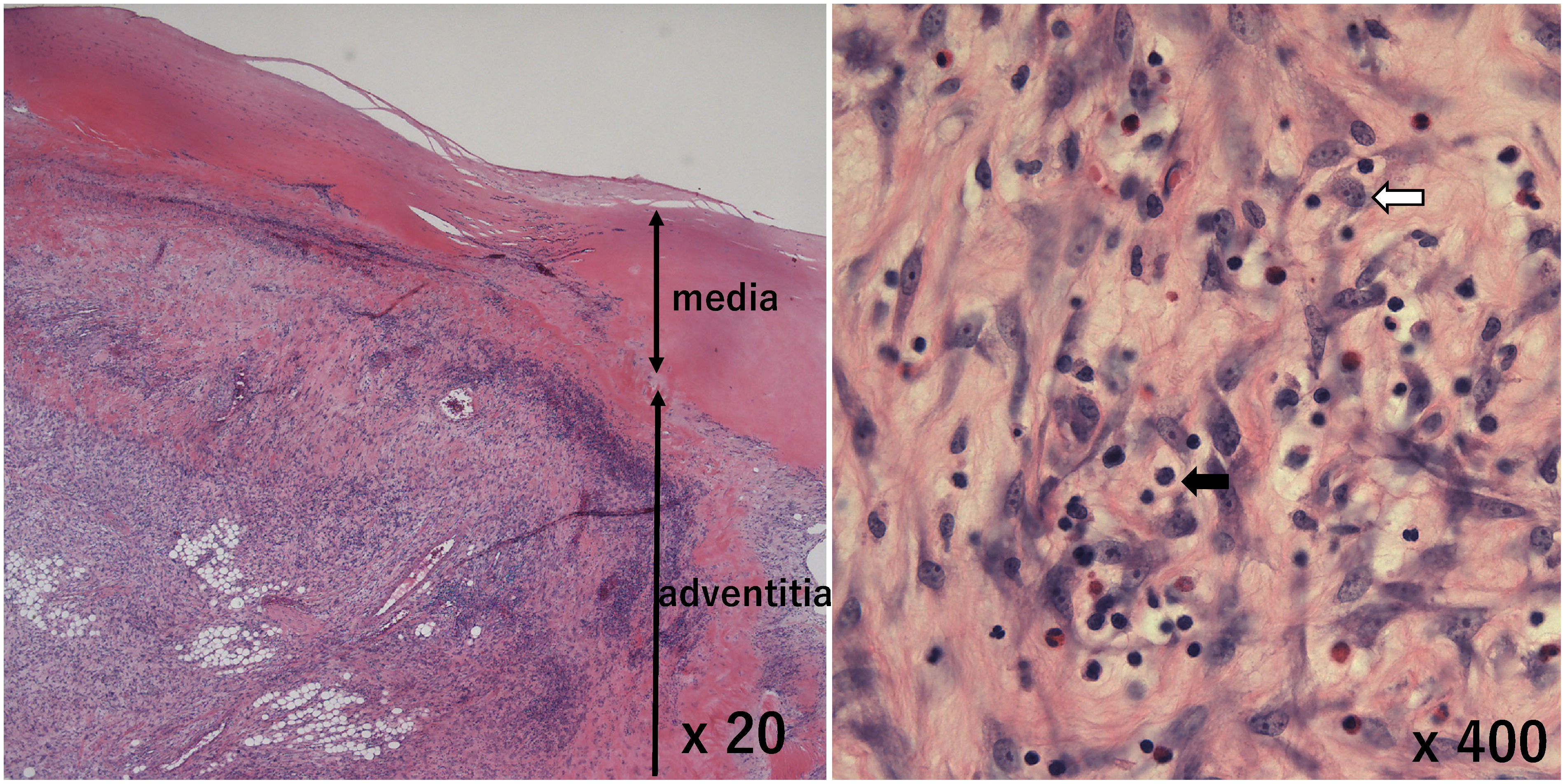
Fig. 3 Microscopic examination findings. Dense infiltration of inflammatory cells in a thickened adventitia as well as adventitial fibrosis and a thin media with decreased elastic fibers were observed (hematoxylin and eosin stain; 20×). Lymphocytes functioning as inflammatory cells (black arrow) and fibroblast cells (white arrow) were also present (hematoxylin and eosin stain; 400×).

The cultures of the aneurysm and adjacent tissue were negative. Histological examination of the aneurysmal wall showed dense infiltration of inflammatory cells, which mainly comprised lymphocytes that were predominantly located in the media and adventitia. Adventitial fibrosis was also observed, and the tunica media exhibited partially decreased, fractured, and absent elastic fibers (**Fig. 3**). Immunohistochemical staining was negative for immunoglobulin G4. Intraoperative and histopathological findings indicated an immunoglobulin G4-unrelated inflammatory aneurysm. The patient’s postoperative course was uneventful. Postoperative CT revealed good patency of the interposed graft. At 2-year follow-up, the patient was alive and well, and contrast-enhanced CT showed no evidence of the occlusion.

## Discussion

Visceral artery aneurysms are rare vascular entities, and most involve the splenic and hepatic arteries. SMAAs are the third most common visceral artery aneurysms, accounting for only 6.9% of all such aneurysms.^1)^ The epidemiology of SMAAs shows male predominance and an average age at onset within the fourth to fifth decade of life.^2,3)^ Most SMAAs affect the proximal ≤5 cm of the SMA.^2)^ Peripheral SMAAs, as in the present case, are uncommon. The clinical symptoms of SMAAs are usually variable and nonspecific, such as abdominal pain or nausea, which mimic gastrointestinal diseases. However, with the advent and current use of cross-sectional imaging, such as ultrasonography and CT, most SMAAs are incidentally detected. Some authors have reported that 48–70% of SMAAs are asymptomatic.^1,2)^ In the present case, the SMAA was also asymptomatic and was incidentally diagnosed during an examination for cholecystitis.

The reported etiologies of SMAAs include the following: infection; atherosclerosis; trauma; dissection; cystic medial dysplasia; fibromuscular dysplasia; segmental arterial mediolysis; collagen vascular disease, including systemic inflammatory disorders such as polyarteritis nodosa; Takayasu’s arteritis; and ANCA-associated vasculitis. Infection was historically thought to be the most common etiology. However, a recent study showed that atherosclerosis was the most common pathological finding and that only 4.7% of patients had an infectious etiology.^1)^ In the present case, the intraoperative and histopathological findings were consistent with typical characteristics of an inflammatory aneurysm: a white and glistening aneurysmal surface, thickened aneurysmal wall, dense adherence of adjacent structures, thinning of medial smooth muscle, adventitial fibrosis, and lymphoplasmacytic infiltration. Lymphoplasmacytic infiltration, which was also found in the peri-adventitia, was presumed to be caused by the spread of aneurysmal inflammation. Therefore, we suspected an inflammatory aneurysm based on these intraoperative and pathological findings, which were similar to the findings of previous reports. Additionally, there was low possibility of systemic vasculitis based on the preoperative findings of negative serum autoantibodies and the absence of other coexisting vascular disorders. Therefore, the inflammation of the SMAA was likely to be associated with a primary localized inflammatory process. To the best of our knowledge, only two reports of so-called inflammatory visceral artery aneurysms have been published to date.^4,5)^ In the present case, as Dorigo et al. also reported, preoperative CT showed no aneurysmal wall thickening, which is a typical finding of an inflammatory aneurysm, contrasting the intraoperative findings.^5)^ We think that there was no relation between cholecystitis and SMAA because the locations of the gallbladder and SMAA on CT images were definitely separate and because tissues between them were intraoperatively found to be intact. Therefore, inflammation due to the cholecystitis was unlikely to have spread only through the SMAA. Furthermore, blood cultures and resected aneurysmal wall cultures were negative, indicating a low possibility of a mycotic aneurysm due to cholecystitis.

SMAAs have a high tendency to rupture. Stone et al. reported a 38% rupture rate at presentation and a 37.5% mortality rate for ruptured aneurysms.^1)^ Due to the potentially life-threatening complications, appropriate management of SMAAs is mandatory to achieve satisfactory clinical outcomes. No definite surgical indication has been established regarding the size of SMAAs that should be treated. Marone et al. recommended repairing SMAAs of >2 cm in diameter,^6)^ while Lee recommended repairing SMAAs of >2.5 cm in diameter.^7)^ However, some authors have reported favorable outcomes of conservative therapy for small asymptomatic SMAAs.^2,3)^ This might indicate that asymptomatic small aneurysms can be followed with serial imaging. However, considering the high rate of SMAA rupture, we believe that early surgical intervention is definitely crucial, and we agree that a diameter of >2 cm (with the exception of symptomatic or mycotic aneurysms) is a reasonable indication for surgical intervention.

Simple ligation is one of the common surgical procedures in treatment of SMAAs. Simple ligation and aneurysmectomy without reconstructing the SMA might be acceptable in patients with potential collateral circulation from the celiac artery or inferior mesenteric artery to the SMA. However, several cases treated with this approach resulted in poor outcomes due to intestinal ischemia.^1,8)^ The necessity of SMA reconstruction depends primarily on the patient’s underlying vascular status and the anatomical site of the aneurysm, which determines the likelihood of ischemia distal to the site following aneurysmal resection. Obara et al. reported that when the aneurysm expands beyond the branch of the inferior pancreaticoduodenal artery or the middle colic artery from the origin of the SMA, the peripheral SMA may become isolated from the collateral circulation routes from the celiac and inferior mesenteric arteries, resulting in intestinal ischemia from reduced blood flow to the SMA.^9)^ Acute bowel ischemia can be fatal. Additionally, according to a review article by Kordzadeh et al., aneurysmectomy alone for mesenteric aneurysms was strongly associated with significant bowel resection, whereas aneurysmectomy with maintenance of peripheral flow demonstrated excellent outcomes.^8)^ Jiang et al. also reported that bowel ischemia occurred secondary to ligation of many small branches despite SMA reconstruction.^2)^ Additionally, although the color of the intestine can appear normal during the operation, postoperative dynamic changes, including dehydration or deterioration of the conditions, indicates a risk of intestinal ischemia if revascularization of the SMA is not performed. Therefore, we always recommend performing aneurysmectomy with reconstruction of the SMA, including its major branches, when it is technically feasible and when the vessels are large enough for an anastomosis. In case of the reconstruction under active inflammation such as infection, there might be a risk for dehiscence of the anastomosis. However, in the present case, there was little risk for dehiscence of the anastomosis because obvious active inflammation findings, such as positive blood cultures, high inflammatory laboratory values, and intraoperative vulnerable tissues, were not observed. Furthermore, it is necessary to carefully evaluate the viability of the intestinal tract during the operation to avoid acute intestinal ischemia. Although changes in the color of the intestine during the operation cannot fully guarantee the safety of the intestine, we believe that this is an effective sign for avoiding intestinal ischemia.

Recently, endovascular repair has proved to be a safe and effective treatment for SMAAs. However, some authors have reported endoleakage resulting from retrograde filling of collateral vessels, inner-stent thrombosis, and ruptures after endovascular repair, sometimes with fatal outcomes.^2,3)^ In addition, the long-term results of endovascular repair for SMAAs remain unknown. Jiang et al. reported that unsuitable conditions for endovascular repair for SMAAs are as follows: many arteries originate from the aneurysm neck or the aneurysm itself, the aneurysm is giant and does not have an adequate landing zone, and the aneurysm has an infectious etiology.^2)^ In the present case, we resected the aneurysms and reconstructed the SMA because we believe that maintaining adequate mesenteric circulations is important.

## Conclusion

SMAAs are rare vascular disorders that are usually diagnosed incidentally, but they may become life-threatening due to their high rupture incidence. Although several surgical techniques are available for SMAAs, we believe that resection of the aneurysm and reconstruction of the superior mesenteric artery is the preferred procedure to maintain adequate mesenteric circulations, including the major branching vessels of the SMA, if feasible. The vascular surgeon should be aware of the possibility of an inflammatory etiology of SMAAs, although this is extremely rare. Histopathological examination is very important in cases of open surgery.

## Additional Note

The patient has provided permission to publish these features of his case, and his identity has been protected.
